# Injury profiles and attitudes toward safety handling skills among emergency medical technicians: an integrated behavioral model analysis

**DOI:** 10.1186/s12889-025-24200-2

**Published:** 2025-08-31

**Authors:** Tzu-Fu Huang, Chung-Chia Chang, Yu-Tung Chang

**Affiliations:** 1 Shin Kong Wu Ho-Su Memorial Hospital, Taipei City, Taiwan; 2 New Taipei City Fire Department, New Taipei City, Taiwan; 3 National Taipei University of Nursing and Health Sciences, Taipei City, Taiwan

**Keywords:** Manual handling, Emergency medical technicians, Health belief model, Transtheoretical model, Musculoskeletal disorders

## Abstract

**Background:**

Manual handling injuries, particularly musculoskeletal disorders (MSDs), are a leading occupational health concern for Emergency Medical Technicians (EMTs) globally, including Taiwan. Lower back injuries are prevalent, often resulting from labor-intensive patient-handling techniques. Despite their critical role, limited training, cultural barriers, and underreporting exacerbate these risks, necessitating targeted interventions.

**Methods:**

This study analyzed manual handling practices among Taiwanese EMTs, focusing on injury prevalence, reporting behavior, training adequacy, and the use of ergonomic equipment. A structured questionnaire was employed. The Health Belief Model (HBM) and Transtheoretical Model (TTM) were applied to examine behavioral factors influencing safe handling practices, including perceived risks, benefits, and barriers.

**Results:**

The findings revealed a high prevalence of MSDs, with lower back injuries being the most common. Injuries occurred, on average, after 5.3 years of service, aligning with international data. Training was identified as insufficient, with Taiwanese EMTs receiving two hours compared to Victoria’s three-day programs. Behavioral analysis using HBM and TTM highlighted self-efficacy and perceived benefits as critical facilitators of safe practices, while perceived barriers and low susceptibility hindered adoption. Additionally, a lack of ergonomic tools and reliance on traditional methods increased injury risks.

**Conclusions:**

The study underscores the urgent need for policy reforms, expanded training programs, and ergonomic equipment adoption to mitigate manual handling injuries among EMTs in Taiwan. Promoting a culture of safety and improving injury reporting mechanisms are essential. These findings provide a foundation for evidence-based interventions to enhance EMT well-being and operational safety.

**Trial registration:**

This project was approved by the Research Ethics Committee of the En Chu Kong Hospital under number ECKIRB1130507. The date of approval is June 12th, 2024.

**Supplementary Information:**

The online version contains supplementary material available at 10.1186/s12889-025-24200-2.

## Background

Emergency Medical Technicians (EMTs) are vital as prehospital care providers. Their working environment often requires frequent manual handling and patient movement, which can result in a high incidence of work-related injuries [1]. Research indicates that the prevalence of work-related injuries and disorders among EMTs ranges from 27% to 45%. Of these, approximately 56.52% are classified as musculoskeletal disorders (MSDs) [2–4]. Studies have demonstrated that frequent manual handling practices significantly contribute to the risk of developing MSDs, with lower back injuries comprising around 47.4% to 70.8% of all MSDs reported [4–6]. Sprains and strains are the most commonly documented injury types within this category. As a result, the challenges associated with manual handling and the high occurrence of MSDs represent critical concerns for occupational health and safety within emergency medical services [1, 7].

In Taiwan, all firefighters are EMTs, also called EMT-Firefighters, who have to take turns in fire rescue shifts and ambulance services shifts every day. The National Fire Agency (NFA) Firefighter Injury and Death Report is the official document addressing EMTs’ occupational health and safety in Taiwan. According to the 2022 NFA report, 45% of injuries occurred during prehospital care, with head injuries and road accidents being the most prevalent types sustained by Taiwanese EMT-Firefighters [2]. Conversely, previous studies have identified musculoskeletal disorders (MSDs) as the leading type of injury affecting EMTs rather than head injuries [2, 4]. This inconsistency between the literature and the NFA report may be since the NFA report does not encompass data on MSDs and manual handling in its survey.

Amit and Hunter (2024) conducted a cross-sectional study in the United States using RULA and REBA assessments to evaluate musculoskeletal disorder (MSD) risks among EMTs during five common patient-handling tasks. The highest risk was observed during ground-to-stretcher lifts, with 43% of EMTs reporting back pain. The study identified posture, load, and force as key risk factors, highlighting the urgent need for ergonomic interventions and training to reduce lower back injuries among EMTs [8]. Additionally, studies focusing on manual handling among healthcare providers in hospitals and home care settings reveal that injuries with lower back injuries make up 77.2% of cases [9]. The absence of manual handling surveys for EMTs in Taiwan highlights a critical gap in current research, making this study essential.

No Lifting Policy (NLP) emphasizes a safer approach to patient handling with manual lifting by using minimized vertical lifting and adequate assistive devices and encouraging patients to use their own muscle strength [10, 11]. Chen et al. (2014) suggested six common scenarios involving high loading and exertion, including rolling, repositioning, sitting up from lying on a bed, transferring from bed to bed, transferring from bed to chair, and rising up from the floor [12]. Currently, there is a lack of research on the manual handling scenarios commonly faced by EMTs in prehospital settings. Additionally, no studies have focused on the educational training or assistive equipment available for these scenarios. This study aims to investigate the typical manual handling practices among EMTs in Taiwan, identify the scenarios that are most likely to result in injuries and propose recommendations for improvement.

Behavioral science theories primarily focus on studying or intervening in the behaviors of specific target groups to achieve desired behavioral changes. Before the implementation of safety manual handling training for Taiwanese EMT-firefighters, the questions that should be addressed include the target audience’s attitude toward adopting safety skills and motivation to learn and apply new skills in real practice. The Health Belief Model (HBM) examines individuals’ attitudes toward performing a target behavior, including perceived susceptibility, perceived severity, perceived benefits, perceived barriers, self-efficacy, and cues to action [13]. Tu et al. studied musculoskeletal disorders (MSDs) and utilized the HBM to investigate related issues [13]. The Transtheoretical Model (TTM) categorizes behavior change into five stages: Pre-contemplation, Contemplation, Preparation, Action, and Maintenance. Since individuals reside in different stages of the TTM at any given time, tailored intervention strategies are required to facilitate progression to the next stage. However, no prior research has applied the TTM to investigate whether EMTs adopt safe manual handling practices. Consequently, the distribution of EMTs across the various TTM stages remains unknown [14].

This study aimed to investigate the prevalence of work-related injuries and MSDs among EMTs by integrating the HBM and the TTM models into a structured questionnaire. This questionnaire was designed to explore EMTs’ attitudes toward adopting safe manual handling practices at various stages of behavioral change, as outlined in the TTM.

## Methods

This was a cross-sectional observation study that administered a structural online questionnaire. The inclusion and exclusion criteria for this questionnaire were carefully designed to ensure the validity and relevance of the study population. Eligible participants were required to be at least 20 years old, currently employed as Emergency Medical Technicians (EMTs) or firefighter-EMTs in Taiwan, and to have actively participated in prehospital patient handling within the past year. Participants needed to be able to read and understand Chinese and to provide informed consent prior to participation. Individuals were excluded if they were administrative or support staff without direct patient-handling duties, students or interns without independent EMT certification, or if they had not engaged in patient handling in the preceding year. Additionally, those unable to comprehend the questionnaire due to language or cognitive barriers, or those on extended leave for major health conditions, were excluded. This approach ensured the study focused on frontline EMTs with recent, relevant experience in manual handling tasks.

The data collection period started from July to September 2024. The official NFA channel and local fire departments administered the questionnaire. To determine the appropriate sample size for this study, we employed a standard formula for sample size estimation in finite populations. The total population size (N) was 20,672, including 16,990 full-time EMT-Firefighters and 3,682 volunteer EMTs. We selected a confidence level of 95% and a margin of error (e) of 5%, which are commonly accepted thresholds in social and health sciences research. Assuming a conservative response distribution (*p* = 0.5) to maximize sample size, the initial calculation was performed using the following formula for an infinite population:

$$n_0=\frac{z^2\cdot p\cdot\left(1-p\right)}{e^2}=\frac{1.96^2\cdot0.5\cdot0.5}{0.05^2}=384.16$$

To adjust for the finite population size, we applied the finite population correction (FPC):

$$n=\frac{n_0}{1+\left({\displaystyle\frac{n_{0-1}}N}\right)}=\frac{384.16}{1+\left({\displaystyle\frac{384.16-1}{20672}}\right)}\approx377.7$$

**Fig. 1:**
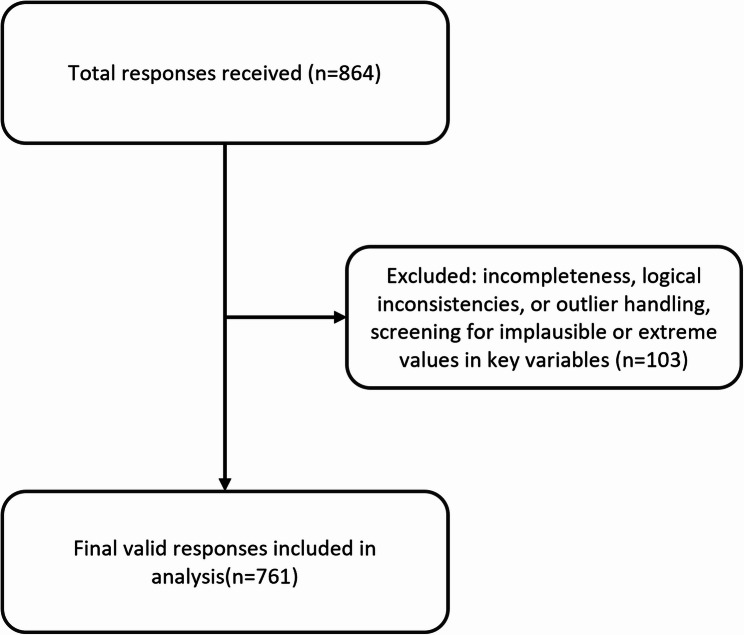
Flowchart of Questionnaire Response Inclusion

Rounding up, the final required sample size was determined to be 378. This calculation ensures that the sample adequately represents the population with the desired precision and confidence level. An additional 20% of participants were included to ensure a valid sample size, bringing the target sample size to 454. Ultimately, a total of 864 responses were received. After excluding 103 responses due to incompleteness, logical inconsistencies, or outlier handling, screening for implausible or extreme values in key variables (e.g., years of experience, number of injuries) was addressed, and such cases were reviewed and excluded if deemed erroneous. 761 valid responses were included in the final analysis. The participant inclusion process is shown in Fig. 1.

The questionnaire (see Supplementary) was designed based on a comprehensive review of the literature and expert input to ensure content validity and relevance to the study objectives. The questionnaire utilized in this study is structured into four distinct sections. The first section gathers demographic and occupational background information, including variables such as gender, age, years of professional experience, geographic work location, professional role, the organizational type of the work unit, frequency of engagement in various manual handling scenarios, and methods of assistive device utilization. The second section investigates participants’ prior injury experiences, including specific anatomical injury sites sustained during duty. The third section, grounded in the TTM, referred to the Health Behaviors and Stages of Change Questionnaire (HBSCQ) to assess participants’ attitudinal stages related to adopting safe manual handling practices, which included the stages of pre-contemplation (have not thought to do), contemplation (plan to do in the next six months), preparation (ready to do in a month), action (have started but still cannot do it regularly), and maintenance (have done it regularly for more than six months) [15]. The fourth section evaluates the constructs of the HBM relevant to the intention to perform safe manual handling techniques, encompassing factors such as perceived susceptibility, perceived severity, perceived benefits, perceived barriers, self-efficacy, and cues to action. The items in the third and fourth sections are developed based on the theoretical frameworks of TTM and HBM. These two sections use a scoring system of a five-point Likert scale, ranging from “strongly agree” (score 5), “agree” (score 4), “neutral” (score 3), “disagree” (score 2), and “strongly disagree” (score 1) is employed to measure participants’ responses.

This study employs a self-developed structured questionnaire. The questionnaire underwent expert validity evaluation by five domain experts, resulting in a Content Validity Index (CVI) of 0.81, indicating a high degree of consistency. The pilot study’s reliability analysis yielded a Cronbach’s alpha coefficient of 0.94, demonstrating excellent internal consistency. Additionally, validity analysis showed a Kaiser-Meyer-Olkin (KMO) measure of sampling adequacy of 0.93, and Bartlett’s Test of Sphericity was statistically significant, confirming the appropriateness of the data for factor analysis. The statistical methods employed in this study include descriptive and inferential analyses. Descriptive statistics will be used to calculate frequencies, percentages, means, and standard deviations. For inferential statistics, correlation coefficients will be computed to examine the relationships among various dimensions of the HBM. Additionally, multinomial logistic regression will be utilized to analyze the effects of demographic variables and HBM attitudinal factors on predicting the stages of behavior change as outlined by the TTM. Statistical analyses will be conducted using IBM SPSS version 29.0. This study has received approval from the Institutional Review Board (IRB) under protocol number ECKIRB1130507.

## Results

A total of 761 participants completed the questionnaire (male 85.7%, female 14.3%). Table 1 provided demographic information from participants. The participants had an average age of 38 years, an average working experience of 11.4 years, an average exercise time of 5.3 h per week, and an average first work-related injury time of 3.17th years. The working region of the participants were mainly from New Taipei City (*n* = 171, 22.5%), Taipei City (*n* = 147, 19.3%), and Taichung City (*n* = 88, 11.6%). The ambulance branch types included general fire and EMS branches (*n* = 588, 77.3%) providing basic life support skills, advanced life support branches (*n* = 148, 19.4%) providing advanced life support skills and paramedical services, and fire and rescue branches (*n* = 25, 3.3%). The education level among participants was mainly undergraduate degrees (*n* = 542, 71.2%). The prevalence of work-related injuries and MSDs among EMTs was also demonstrated in Table 1. 108 participants (14.2%) had been diagnosed with skeletomuscular injuries or illness before joining the services. Over half of the Participants suffered injuries from both fire and EMS duties (*n* = 394, 51.8%). The most frequently injured body part among prehospital personnel was the lower back (*n* = 375, 18.1%), followed by the wrist (*n* = 226, 10.9%) and palm (*n* = 217, 10.5%).

**Table 1:** Demographics and injury-on-duty experiences among participants (*N* = 761)

Variables	*n*(%)	Mean(SD)
Age		38.0(8.3)
Working experiences (Year)		11.4(8.0)
Average weekly exercise time (Hours)		5.3(10.3)
In which year did you experience the first work-related injury as a prehospital care personnel?		3.17(3.17)
Gender		
Male	652(85.7)	
Female	109(14.3)	
Working Region		
New Taipei City	171(22.5)	
Taipei City	147(19.3)	
Taichung City	88(11.6)	
Taitung County	63(8.3)	
Taoyuan City	48(6.3)	
Miaoli County	40(5.3)	
Kaohsiung City	55(7.2)	
Changhua County	26(3.4)	
Tainan City	15(2.0)	
Nantou County	20(2.6)	
Hsinchu City	22(2.9)	
Others	66(8.7)	
Ambulance branch type		
General fire and EMS branch	588(77.3)	
Fire branch with ALS team	148(19.4)	
Search and rescue	25(3.3)	
Education		
Senior high school or below	73(9.6)	
Undergraduate degree	542(71.2)	
Postgraduate degree	146(19.2)	
Diagnosed any skeletomuscular injuries before joining the service		
Yes	108(14.2)	
No	653(85.8)	
Have you ever been injured on duty?		
Never	171(22.5)	
Fire rescue only	133(17.5)	
EMS only	63(8.3)	
Both fire rescue and EMS	394(51.8)	
Most frequently injured body part		
Head	85(4.1)	
Neck	36(1.7)	
Chest	22(1.1)	
Abdomen	23(1.1)	
Upper back	101(4.9)	
Lower back	375(18.1)	
Upper arm	90(4.3)	
Forearm	136(6.6)	
Elbow	99(4.8)	
Wrist	226(10.9)	
Palm	217(10.5)	
Thigh	70(3.4)	
Calf	116(5.6)	
Knee	213(10.3)	
Ankle	177(8.6)	
Sole	51(2.5)	
Pelvis	32(1.5)	

Table 2 summarizes the results of the patient and manual handling of related injuries among prehospital care personnel. The patient handling-related injuries occurred in the average of 5.3 years in 76.9% of participants. The recovery time from the injuries required 6.2 weeks. On average, the patient handling skills required 2 prehospital care personnel to deliver in actual practice. The patient’s weight that caused prehospital care personnel injury was an average of 91.3 kg. The average patient handling moving distance was 2.8 floors vertically, which caused injury. The types of patient handling-related injuries included strain/sprain (88.4%), abrasions (3.5%), blunt injury (2.6%), fracture (2.0%), cuts/lacerations (1.4%), and other injuries (2.0%). The results also revealed that lower back injury represents the highest percentage of injuries (65.6%), followed by wrist injury (7.1%) among prehospital care personnel. The most frequent causes of patient handling-related injury were 2-person manual handling (40.9%) and Evac-mat (30.5%). Self-care with (42.2%) and without (54.8%) medical attention were the main options for personnel after injury. 97.1% of injuries were not reported to managers or organizations. When handling a patient, 54.4% of the time, personnel were carrying other equipment. According to the level of consciousness, 34.4% of patient-handling-related injuries were unresponsive, and 31.0% were alert patients.

**Table 2:** Results of patient-handling-related injuries

Experience	*n*(%)	Mean(SD)
In which year of service did this most severe injury from moving a patient occur?		5.3(6.3)
How many weeks did it take for you to feel fully recovered after the injury?		6.2(10.5)
At the time of the injury, how many people were involved in the patient transfer (including yourself)?		2.0(0.9)
What was the approximate weight of the patient during the injury?		91.3(26.9)
How many floors were vertically moved during the injury?		2.8(4.7)
Have you ever been injured while moving or handling a patient?		
Yes	454(76.9)	
No	136(23.1)	
What was the most severe injury you sustained from moving a patient?		
Fracture	10(2.0)	
Strain/Sprain	434(88.4)	
Abrasions	17(3.5)	
Blunt trauma	13(2.6)	
Cuts/lacerations	7(1.4)	
Penetration injury	1(0.2)	
Burn injury	2(0.4)	
Chemical burns	1(0.2)	
Others	6(1.2)	
Where was the most severe injury located?		
Head	9(1.8)	
Neck	4(0.8)	
Chest	3(0.6)	
Abdomen	10(2.0)	
Upper back	16(3.1)	
Lower back	333(65.6)	
Upper arm	13(2.6)	
Forearm	12(2.4)	
Elbow	7(1.4)	
Wrist	36(7.1)	
Palm	14(2.8)	
Thigh	4(0.8)	
Calf	2(0.4)	
Knee	9(1,8)	
Ankle	23(4.5)	
Sole	2(0.6)	
Pelvis	10(2.0)	
What method of transport were you using during the injury?		
Manual lifting (2 person)	199(40.9)	
Spinal board	60(12.3)	
Evacuation sheet	148(30.5)	
Manual lifting (1 person)	13(2.7)	
Assistive devices	38(7.8)	
Stretcher	27(5.6)	
How was your injury treated?		
Self-care without medical attention	274(54.8)	
Self-sought medical care	211(42.2)	
Medical care with assistance from colleagues or friends	15(3.0)	
Did you file a report related to the workplace injury after the incident?		
Yes	17(2.9)	
No	379(64.2)	
Not meeting the criteria for reporting	194(32.9)	
Did the injury from the duty significantly impact your work?		
Severe impact	49(9.7)	
Moderate impact	169(33.4)	
Slight impact	236(46.6)	
No impact	52(10.3)	
Were you carrying other equipment during the injury?		
Yes	272(54.4)	
No	228(45.6)	
What was the patient’s level of consciousness during the injury?		
Alert	155(31.0)	
Response to voice	89(17.8)	
Response to Pain	84(16.8)	
Unresponsive	172(34.4)	

Table 3 summarizes the distribution of participants in the behavioral change stages based on the transtheoretical model and their perceived attitude toward adopting safety manual handling skills within the behavioral change stages. Most participants were in the Contemplation stages (45.5%) and Pre-contemplation (18.8%). The Maintenance stage occupied 16.3% of the participants who had already adopted the skills for more than 6 months. Among the five behavioral change stages, perceived severity and perceived benefit were the highest attitude levels in adopting safety manual handling skills. This reflects that the prehospital care personnel acknowledge the severe consequences of not adopting safety manual handling skills and the benefits of adopting the skills in practice. In the pre-contemplation stage, the lowest behavioral attitude was the cues to action, which means the participants in this stage require more information or hints to action. The lowest behavioral attitude in the contemplation, preparation, action, and maintenance stages was the perceived barriers, which indicated that the participants acknowledged fewer barriers in adopting safety manual handling skills.

**Table 3:** Cross-table between HBM factors and stages of adopting safety patient handling skills (*N* = 761)

	Precontemplation	Contemplation	Preparation	Action	Maintenance
	*n* = 143(18.8%)	*n* = 346(45.5%)	*n* = 117(15.4%)	*n* = 31(4.0%)	*n* = 124(16.3%)
	Mean(SD)	Mean(SD)	Mean(SD)	Mean(SD)	Mean(SD)
Perceived susceptibility	3.82(0.95)	3.86(0.76)	3.77(0.86)	3.70(0.90)	3.35(0.99)
Perceived severity	4.06(0.83)	4.16(0.69)	4.08(0.75)	4.29(0.60)	4.05(0.80)
Perceived benefits	4.02(0.89)	4.31(0.61)	4.28(0.71)	4.50(0.63)	4.36(0.70)
Perceived barriers	3.76(0.89)	3.64(0.77)	3.45(0.87)	3.39(0.63)	3.19(0.85)
Self-efficacy	3.84(0.84)	3.81(0.64)	3.93(0.57)	3.84(0.57)	3.99(0.62)
Cues to action	3.64(0.90)	3.67(0.73)	3.86(0.64)	3.85(0.51)	3.87(0.72)

The stages of adopting safety manual handling skills positively correlate with perceived benefit, self-efficacy, and cues to action with statistical significance (see Table 4). These results indicate that during the stages of behavioral change toward action, the participants develop stronger attitudes toward perceived benefit, self-efficacy, and cues to action. However, the stages of adopting safety manual handling skills have a negative relationship with perceived susceptibility and perceived barriers, which indicates that during the stages of behavioral change toward action, the participants feel fewer attitudes toward perceived susceptibility and perceived barriers (see Table 4).

**Table 4:** The relationship between HBM factors and behavioral change stages

	Stages of adopting safety handling	Perceived susceptibility	Perceived severity	Perceived benefits	Perceived barriers	Self-efficacy	Cues to action
Stages of adopting safety handling	1						
Perceived susceptibility	− 0.182^**^	1					
Perceived severity	− 0.014	0.648^**^	1				
Perceived benefits	0.121^**^	0.293^**^	0.518^**^	1			
Perceived barriers	− 0.224^**^	0.563^**^	0.430^**^	0.221^**^	1		
Self-efficacy	0.085^*^	0.356^**^	0.416^**^	0.492^**^	0.383^**^	1	
Cues to action	0.116^**^	0.243^**^	0.287^**^	0.402^**^	0.402^**^	0.623^**^	1

**P < 0.01

*P < 0.05

Tables 5 and 6 show the results of multinomial logistic regression with stages of adopting safety manual handling as a dependent variable and HBM factors as independent variables, including perceived susceptibility, perceived severity, perceived benefits, perceived barriers, self-efficacy, and cues to action. McFadden’s Pseudo R-square in this model was 0.068, indicating that the model has a relatively lower fit for predicting our outcome categories. The likelihood ratio tests indicate that perceived susceptibility, perceived benefits, perceived barriers, self-efficacy, and cues to action significantly improve model fit and strongly predict the outcome variable. However, perceived severity was not significant (chi-square = 4.88, df = 4, *p* = 0.299), indicating that removing perceived severity does not significantly decrease model fit (see Table 5).

**Table 5:** Likelihood ratio test results for predictors in the multinomial logistic regression model

Effect	Model Fitting Criteria	Likelihood Ratio Tests		
	−2 Log Likelihood of Reduced Model	Chi-Square	df	Sig.
Intercept	1712.208	10.011	4	0.040
Perceived susceptibility	1721.280	19.083	4	0.001
Perceived severity	1707.081	4.884	4	0.299
Perceived benefits	1724.030	21.833	4	0.000
Perceived barriers	1745.986	43.789	4	0.000
Self-efficacy	1717.748	15.551	4	0.004
Cues to action	1720.280	18.083	4	0.001

The chi-square statistic is the difference in −2 log-likelihoods between the final model and a reduced model. The reduced model is formed by omitting an effect from the final model. The null hypothesis is that all parameters of that effect are 0

**Table 6:** Parameter estimates for the multinomial logistic regression model

		B	Std. Error	Wald	df	Sig.	Exp(B)	95% Confidence Interval for Exp(B)	
								Lower Bound	Upper Bound
Contemplation	Intercept	−0.281	0.697	0.162	1	0.687			
	Perceived susceptibility	0.158	0.190	0.688	1	0.407	1.171	0.806	1.701
	Perceived severity	0.076	0.222	0.116	1	0.733	1.079	0.699	1.665
	Perceived benefits	0.814	0.191	18.243	1	0.000	2.258	1.554	3.281
	Perceived barriers	−0.458	0.191	5.747	1	0.017	0.633	0.435	0.920
	Self-efficacy	−0.612	0.236	6.736	1	0.009	0.542	0.342	0.861
	Cues to action	0.243	0.192	1.599	1	0.206	1.275	0.875	1.858
Preparation	Intercept	−1.393	0.897	2.412	1	0.120			
	Perceived susceptibility	0.207	0.233	0.790	1	0.374	1.230	0.780	1.940
	Perceived severity	−0.028	0.271	0.011	1	0.917	0.972	0.571	1.654
	Perceived benefits	0.508	0.235	4.656	1	0.031	1.662	1.048	2.636
	Perceived barriers	−1.042	0.222	22.013	1	0.000	0.353	0.228	0.545
	Self-efficacy	−0.204	0.289	0.498	1	0.480	0.816	0.463	1.436
	Cues to action	0.796	0.246	10.479	1	0.001	2.216	1.369	3.588
Action	Intercept	−4.351	1.735	6.291	1	0.012			
	Perceived susceptibility	−0.182	0.317	0.332	1	0.565	0.833	0.448	1.550
	Perceived severity	0.587	0.408	2.069	1	0.150	1.799	0.808	4.005
	Perceived benefits	1.100	0.412	7.116	1	0.008	3.004	1.339	6.739
	Perceived barriers	−1.012	0.313	10.428	1	0.001	0.364	0.197	0.672
	Self-efficacy	−0.762	0.402	3.604	1	0.058	0.467	0.212	1.025
	Cues to action	0.791	0.356	4.949	1	0.026	2.206	1.099	4.428
Maintenance	Intercept	−1.485	0.959	2.401	1	0.121			
	Perceived susceptibility	−0.518	0.217	5.668	1	0.017	0.596	0.389	0.913
	Perceived severity	0.365	0.261	1.961	1	0.161	1.440	0.864	2.400
	Perceived benefits	0.630	0.243	6.699	1	0.010	1.877	1.165	3.025
	Perceived barriers	−1.184	0.221	28.792	1	0.000	0.306	0.199	0.472
	Self-efficacy	0.119	0.290	0.167	1	0.683	1.126	0.638	1.987
	Cues to action	0.749	0.244	9.416	1	0.002	2.114	1.311	3.410

Table 6 presents the estimated coefficients (B), odds ratios (OR), and 95% confidence intervals (CI) for each predictor across the stages of adopting safety manual handling. The stage of pre-contemplation is set as the reference category. The perceived susceptibility and perceived severity have no statistically significant effect on the likelihood of being in stages of contemplation, preparation, action, and maintenance compared to the pre-contemplation outcomes. The multinomial logistic regression analysis results indicated that compared to the Pre-contemplation stage, participants with higher levels of Perceived benefits, lower levels of Perceived barriers, and lower levels of Self-efficacy were more likely to enter the Contemplation stage; participants with higher levels of Perceived benefits, lower levels of Perceived barriers, and higher levels of Cues to action were more likely to enter the Preparation stage and Action stage; participants with lower levels of Perceived susceptibility, higher levels of Perceived benefits, lower levels of Perceived barriers, and higher levels of Cues to action were more likely to enter the Maintenance stage.

## Discussion

This study represents the first systematic investigation in Taiwan addressing manual handling injuries among EMTs. Previous research has documented that one in four paramedics in Victoria, Australia, experience MSDs within an average of five years of employment [16]. Consistent with these findings, the present study identified an average injury occurrence time of 5.3 years among Taiwanese EMTs, indicating that similar work environments and job demands may contribute to such injuries [2]. To mitigate these injuries, targeted interventions focusing on education, training, and implementing assistive devices are essential [17]. To date, the lack of research has definitively determined the optimal content, duration, or training frequency required to reduce the occurrence of MSDs effectively. However, a notable disparity exists when comparing the manual handling training provided to paramedics and EMTs in Victoria and Taiwan. In Victoria, paramedics are mandated to complete three days (up to 24 h) of manual handling training, whereas Taiwan’s statutory EMT curriculum allocates only two hours of lectures for all levels of EMTs [16, 18]. The major difference is that the “No lift policy” is embedded in the organization’s culture in Ambulance Victoria, so they need to teach more skills and equipment use to minimize the manual lifting behavior; however, the current Taiwanese EMT training is more focused on fast transport and patient movement only. This considerable discrepancy underscores the need for comprehensive research and policy reform to enhance training frameworks for Taiwanese EMTs.

The study revealed that the lower back, palms, and wrists were the most frequently injured body regions during prehospital care. Lower back pain and strains were particularly prevalent during manual handling tasks, corroborating findings from international studies [4, 7, 19]. Specifically, 65.6% of Taiwanese EMTs reported lower back injuries, significantly higher than the 47.3% reported in another research [4]. Detailed analysis indicated that the most frequently employed techniques, including the two-person manual lifting and evacuation sheet, are labor-intensive and primarily reliant on manual effort. Notably, these methods are typically performed without assistive devices in Taiwan. It is critical to evaluate the feasibility of integrating assistive tools into manual handling practices to address this issue. For instance, using devices such as walk assist belts and quick-release straps is standard during manual handling tasks in Victoria [16]. These tools help distribute and reduce direct and vertical forces exerted on the body, particularly the lower back, thereby mitigating injury risk [16]. Incorporating similar practices in Taiwan could alleviate the physical burden on EMTs and reduce injury rates.

This study also found that over 60% of Taiwanese EMTs reported experiencing injuries during prehospital care operations—a proportion notably higher than that documented in the NFA annual workplace injury reports. Most injured EMTs opted for self-care or medical attention without formally reporting their injuries, leading to an underrepresentation of injury statistics in the NFA’s data. In this study, 97% of EMTs with injury experiences did not file official reports. This reporting gap may stem from workplace cultural norms of Taiwan’s firefighting and emergency medical service systems. Addressing this issue will require further investigation into the underlying causes and developing strategies to encourage injury reporting, which could inform future workplace safety policies and practices.

This study identified a strong correlation between the execution of safe handling practices by EMTs and the levels of perceived benefits, perceived barriers, and cues to action. The possible reason why perceived severity was not statistically significant is that it is the weakest predictive factor compared to other HBM factors [17]. When EMTs recognize the long-term benefits of safely handling their personal health and work performance, the likelihood of adopting safe behaviors increases significantly. Conversely, reducing perceived barriers, such as using ergonomic assistive devices, promotes implementing safety practices [16]. Additionally, adequate resources and professional skill training effectively decrease perceived barriers, enhancing the likelihood of behavioral adoption [9]. Clear and actionable cues, such as explicit hand gestures or signage in real-world scenarios, substantially improve execution efficiency [15]. Future efforts should emphasize ergonomic interventions, such as lifting aids and hands-on training programs, to highlight the benefits of correct techniques, mitigate perceived barriers, and provide supportive tools. Such measures will encourage EMTs to adopt safe handling behaviors and to reduce health risks [4, 15].

The results indicate that perceived benefit, perceived barrier, and cue to action are the three HBM factors related to taking safety manual handling action across the TTM stages. This is essential information for us to design the contents of coaching interventions based on the purpose of increasing perceived benefit, decreasing perceived barrier, and providing more cues to action. This study also revealed that over 60% of EMTs remain in the pre-contemplation and contemplation stages, having not yet implemented safe handling practices. This is primarily attributed to insufficient action cues, such as inadequate equipment and training [16]. The results support our initial assumptions about EMTs’ attitudes toward safety practice that re-modeling the training course and raising the awareness of safety manual handling might be the priority for the fire and ambulance authority now. Integrating the HBM and the TTM, this study highlighted the effectiveness of HBM in analyzing and improving EMTs’ handling practices and the utility of TTM in providing stage-specific interventions.

In the pre-contemplation stage, health lectures and case studies helped individuals recognize the importance of behavior change [5]. Barriers to change can be addressed through case interviews or group discussions to build confidence in action feasibility [6]. Providing clear cues to action enabled individuals to consider practical steps toward behavior change [15]. Systematic ergonomic interventions, such as walking assist belts, combined with skill practices and knowledge-sharing, reduced physical strain and enhanced confidence and capability in safe handling practices [7, 15]. Additionally, policy reforms emphasizing “no-lift” methods and the integration of standardized training modules can reduce injury risks [7, 19]. Creating a supportive environment with adequate resources and education will empower EMTs to adopt safe handling practices, reduce the likelihood of musculoskeletal disorders (MSDs) and other injuries, and improve emergency response efficiency.

This study, the first in Taiwan to investigate manual handling injuries among EMTs, has several limitations. The reliance on self-reported data may introduce recall bias or underreporting, particularly for minor or undocumented injuries. As a cross-sectional study, it describes injury prevalence and attitudes toward safety practices but cannot establish causal relationships. Additionally, the sample was drawn from a limited geographic region, which may limit the generalizability of the findings. Key factors such as individual health status, workload, and organizational culture were not extensively explored, potentially introducing unmeasured confounders. For example, the influence of organizational culture, including factors such as a blame culture and fear of complaints, leads EMTs to adopt conservative operational practices in order to avoid potential issues. This, in turn, affects their decision-making and may discourage the adoption of safe handling behaviors. Future research should employ longitudinal designs to understand causal relationships better, include objective measures to enhance data reliability, and expand the sample size and geographic scope for broader applicability. Moreover, examining workplace conditions and policy interventions could provide more comprehensive insights into strategies for preventing manual handling injuries among EMTs.

## Conclusions

This study highlights the critical issue of manual handling injuries among EMTs in Taiwan, where MSDs, particularly lower back injuries, are highly prevalent. The observed average injury occurrence of 5.3 years aligns with international research, suggesting shared risks across similar work environments. The study identified perceived benefits, self-efficacy, and cues to action as key motivators for transitioning through the behavioral change stages, while perceived barriers and susceptibility hindered progress. These findings highlight the need for targeted interventions, such as enhanced training programs and assistive device implementation, to facilitate safer practices and reduce injury rates. This research lays the groundwork for addressing manual handling challenges among EMTs, advocating for evidence-based policies and interventions to enhance occupational health and safety in Taiwan’s emergency medical services. Future studies should explore injury reporting barriers and optimal training protocols to advance this critical area of research further.

## Supplementary Information

Supplementary Material 1.

## Data Availability

The data supporting this study’s findings are available on request from the corresponding author.
