# Supplementary material for: Injury profiles and attitudes toward safety handling skills among emergency medical technicians: an integrated behavioral model analysis
Source: BMC Public Health. 2025 Aug 31;25:2986. doi: 10.1186/s12889-025-24200-2 (PMC12400533; doi:10.1186/s12889-025-24200-2)
Supplement: Supplementary file 1 — Supplementary Material 1. [file 12889_2025_24200_MOESM1_ESM.docx]

**Questionnaire on Factors Associated with Injuries During Patient Transfers and Manual Handling Tasks Among Emergency Medical Personnel**

| Dear Emergency Medical Personnel,  This questionnaire investigates the current status and associated factors of injuries sustained by emergency medical personnel during patient transfers and manual handling tasks while on duty. Below are two important definitions:  **"Manual handling tasks"** are defined as any actions requiring physical exertion, including lifting, pushing, pulling, gripping, throwing, and carrying.  **"Safe patient transfers and manual handling "** refer to tasks performed using ergonomically correct and energy-efficient postures, movements, assistive devices, and equipment, considering the patient’s varying levels of self-support.  Please respond based on your work experience and observations. Your answers will help us understand the prevalence of injuries during patient transfers and manual handling, providing valuable insights for future training programs and procedural improvements. Before completing the questionnaire, carefully read each question and select the answer that best reflects your situation.  Your responses will be used exclusively for academic research, and we kindly request your honest input. All data will be kept confidential and used solely for research purposes. Thank you for taking the time to participate in this study.  Dr. Yu-Tung Chang  Assistant Professor  National Taipei University of Nursing and Health Sciences |
| --- |

1. **Demographics and injury-on-duty experiences among participants**
   1. Your age: ______
   2. Working experience (year): ________
   3. Average weekly exercise time (Hours): ________
   4. In which year did you experience the first work-related injury as a prehospital care personnel? ______
   5. Gender: □Male □Female
   6. Working region: _____________
   7. Serving ambulance branch type: □General fire and EMS branch □Fire branch with ALS team □Search and rescue
   8. Education level □Senior high school or below □Undergraduate degree □Postgraduate degree
   9. Diagnosed any skeletomuscular injuries before joining the service □Yes □No
   10. Have you ever been injured on duty? □Never □Fire rescue only □EMS only □Both fire rescue and EMS
   11. Most frequently injured body part: □Head □Neck □Chest □Abdomen □Upper back □Lower back □Upper arm □Forearm □Elbow □Wrist □Palm □Thigh □Calf □Knee □Ankle □Sole □Pelvis
2. **Patient-handling-related injuries**
   1. In which year of service did this most severe injury from moving a patient occur? _____
   2. How many weeks did it take for you to feel fully recovered after the injury? _______
   3. At the time of the injury, how many people were involved in the patient transfer (including yourself)? _____
   4. What was the approximate weight of the patient during the injury? _____
   5. How many floors were vertically moved during the injury? ______
   6. Have you ever been injured while moving or handling a patient? □Yes □No
   7. What was the most severe injury you sustained from moving a patient? □Fracture □Strain/Sprain □Abrasions □Blunt trauma □Cuts/lacerations □Penetration injury □Burn injury □Chemical burns □Others
   8. Where was the most severe injury located? □Head □Neck □Chest □Abdomen □Upper back □Lower back □Upper arm □Forearm □Elbow □Wrist □Palm □Thigh □Calf □Knee □Ankle □Sole □Pelvis
   9. What method of transport were you using during the injury? □Manual lifting (2 person) □Spinal board □Evacuation sheet □Manual lifting (1 person) □Assistive devices □Stretcher
   10. How was your injury treated? □Self-care without medical attention □Self-sought medical care □Medical care with assistance from colleagues or friends
   11. Did you file a report related to the workplace injury after the incident? □Yes □No □Not meeting the criteria for reporting
   12. Did the injury from the duty significantly impact your work? □Severe impact □Moderate impact □Slight impact □No impact
   13. Were you carrying other equipment during the injury? □Yes □No
   14. What was the patient's level of consciousness during the injury? □Alert □Response to voice □Response to Pain □Unresponsive
3. **Have you ever performed “Safe patient transfers and manual handling “ during EMS operation?**

□I have never considered it, nor do I plan to in the next six months.

□I have considered it and may plan to in the next six months.

□I have considered it and should start preparing in the near future.

□I have already started but have been doing it for less than six months.

□I have been consistently doing it for more than six months.

1. **Your attitude toward operating “Safe patient transfers and manual handling”**

| **Items** | **Strongly Agree** | **Agree** | **Neutral** | **Disagree** | **Strongly Disagree** |
| --- | --- | --- | --- | --- | --- |
| 1. I believe the current scope of my work easily causes occupational injuries. 2. I believe the current work patterns easily lead to occupational injuries. 3. I believe I have already suffered work-related injuries that affect my job. 4. I believe the techniques and equipment I currently use in patient transfer and manual handling pose high risks of work-related injuries. |  |  |  |  |  |
| 1. I believe injuries sustained during duty have severe physical consequences. 2. I believe injuries sustained during duty have serious psychological and emotional impacts. 3. I believe injuries sustained during duty significantly affect daily life. 4. I believe injuries sustained during duty greatly impact job performance. 5. I believe injuries sustained during duty are likely to disrupt my career. |  |  |  |  |  |
| 1. I believe learning and practicing patient transfer and manual handling techniques can prevent injuries. 2. I believe learning and practicing patient transfer and manual handling techniques can reduce the severity of potential injuries. 3. I believe learning and practicing patient transfer and manual handling techniques can prevent patient injuries. 4. I believe learning and practicing patient transfer and manual handling techniques demonstrate professionalism. 5. I believe learning and practicing patient transfer and manual handling techniques enhance work efficiency. |  |  |  |  |  |
| 1. I believe time pressure on-site may delay transportation when performing patient transfer and manual handling. 2. I believe carrying additional equipment to perform patient transfer and manual handling safely is cumbersome. 3. I believe limited manpower on-site hinders the proper execution of patient transfer and manual handling. 4. I believe effective or good communication with patients or their families is challenging during patient transfer and manual handling. 5. I believe transferring and handling critical patients (e.g., unconscious or comatose) is difficult. 6. I believe collaborating with colleagues to perform patient transfer and manual handling is challenging. |  |  |  |  |  |
| 1. I believe I have the ability to safely perform patient transfer and manual handling techniques and use related equipment. 2. I believe I can effectively communicate with patients' families during patient transfer and manual handling. 3. I believe I can guide colleagues in performing patient transfer and manual handling. 4. I believe I can better protect myself at work through increased awareness and regular strength training |  |  |  |  |  |
| 1. Past injuries to myself or colleagues during patient transfer and manual handling have heightened my awareness of injury prevention. 2. I have learned techniques to prevent injuries caused by patient transfer and manual handling through institutional training. 3. I have learned techniques to avoid injuries caused by patient transfer and manual handling by consulting senior staff. 4. I have learned techniques to avoid injuries caused by patient transfer and manual handling by consulting healthcare professionals. |  |  |  |  |  |
